# Unc-5 homolog B (UNC5B) is one of the key downstream targets of N-α-Acetyltransferase 10 (Naa10)

**DOI:** 10.1038/srep38508

**Published:** 2016-12-02

**Authors:** Huiyu Xu, Yong Han, Bing Liu, Rong Li

**Affiliations:** 1 Department of Obstetrics and Gynecology, Reproductive Medical Center, Peking University Third Hospital, Beijing, China; 2 Department of pathology, Zhejiang provincial people’s hospital, Hangzhou, Zhejiang Province, P.R. China; 3 307-Ivy Translational Medicine Center, Laboratory of Oncology, Affiliated Hospital of Academy of Military Medical Sciences, Beijing, China

## Abstract

N-α-acetyltransferase 10 (Naa10) displays alpha (N-terminal) acetyltransferase activity. It functions as a major modulator of cell growth and differentiation. Until now, a few downstream targets were found, but no studies have concerned about which gene is the early event of Naa10 downstream target. As we know, the earlier events may play more significant role in Naa10 pathway. Through construction of Naa10 stably knocked down H1299 cell line, we discovered cell morphological changes induced by Naa10. Moreover, potential function of Naa10 in cell morphogenesis was also indicated using cDNA microarray analysis of the Naa10 stably knock-down cell line. We further discovered that netrin-1 (NTN1) and its receptor UNC-5 Homology B (UNC5B) were the early event among the genes involved in Naa10 stably knocked down induced genes expression changes in cell morphogenesis. This was further validated in caudal half region of E10 mouse embryos. Negative regulation of Naa10 towards NTN1 and its receptor UNC5B were also detected upon treatment of *all-trans* retinoid acid, which was often used to induce morphological differentiation.

N-α-acetyltransferase 10 (Naa10), is the catalytic subunit of N-acetyltransferase A (NatA), catalyzes N-α-acetylation, ε-acetylation, as well as autoacetylation^1^,^2^,^3^,^4^. Naa10 protein is a major modulator of cell growth and differentiation that has been recently reported to be important in cancer progression. Mutations in Naa10 gene in yeast results in defect of cell cycle progression and mating^5^,^6^,^7^. Similarly, deficiency of Naa10 is related to abnormal oogenesis and X-linked lethality in drosophila and human male infants^8^,^9^. In human cells, emerging evidence demonstrate that increased expression of Naa10 protein is associated with tumorigenesis^4^,^10^,^11^,^12^,^13^,^14^,^15^. Furthermore, Naa10 protein suppresses amyloid-beta protein secretion, while accumulation of successive amyloid-beta is responsible for development of a neurodegenerative disorder called Alzheimer’s disease^16^. Moreover, Naa10 is reported to be high expressed in developing mouse brain^17^,^18^, and attenuates when neurons differentiate^18^.

UNC-5 Homolog B (UNC5b), a dependence receptor of netrin-1, plays an essential role in mediating the repulsive effect of axonal migration and blood vessel formation through association with its ligand netrin-1 (NTN1)^19^,^20^. In addition, UNC5B has also been indicated as a putative tumor suppressor gene in numerous cancers, including colorectal, ovary, uterus, stomach, lung or kidney cancers^21^. Despite the significant roles Unc5 receptors exert, currently there is limited knowledge about UNC5B’s binding partners and upstream modulators.

In this study, we first discovered the morphological changes through construction of Naa10 stably down expressed H1299, a non-small cell lung carcinoma cell line. cDNA microarray of Naa10 gene function also indicated the morphogenesis role of Naa10. Tube formation is a reflection of vasculogenesis or angiogenesis, increased tube formation ability was observed upon Naa10 down expression. Measuring the different time points of gene expression upon Naa10 siRNA treatment, NTN1 and its receptor UNC5B was found to be the most dramatically over-expressed among the genes involved in morphogenesis. They were also found to be over-expressed in caudal half region of mouse E10 embryos upon Naa10 siRNA treatment. Besides, the increased mRNA expression of Naa10 and decreased expression of NTN1 and UNC5B were observed upon 24 hours treatment of retinoic acid, a morphological differentiation inducer^22^,^23^,^24^, the expression pattern was reversed after transiently transfection of Naa10 siRNA, which is also an evidence of negative regulation of Naa10 towards NTN1 and its receptor UNC5B.

## Materials and Methods

### Materials

RPMI 1640 medium, DMEM medium and the fetal bovine serum were purchased from Hyclone (UT, USA). The all-*trans* retinoic acid (ATRA) was purchased from Sigma-Aldrich (Saint Louis, MO). G418 antibiotic was purchased from AMRESCO (OH, USA). The human non-small-cell lung carcinoma cell line H1299 was obtained from ATCC (the Global Bioresource Center), supplemented with 10% heat-inactivated fetal bovine serum (FBS) in RPMI1640. The mouse immortalized embryonic endothelial cells, which were established as documented previously^25^, were cultured by DMEM, in addition of 15% FBS. Glyceraldehyde 3-phosphate dehydrogenase (GAPDH) antibody was purchased from Proteintech Group Inc. (IL, USA), while the Naa10 antibody was generated in our own lab^10^. The Enhanced Chemiluminescence Substrate was purchased from Pierce Co. (IL, USA).

### Isolation of embryos and embryonic cell culture

All embryos were obtained from wild-type C57 female mice. The appearance of vaginal plug observation was designated as embryonic day 0 (E0). At E10 (30 somites), female mice were sacrificed, followed by removal of embryos, the caudal half region was then obtained. The tissues were washed with PBS twice, and treated with 0.2% trypsin at 37 °C for 10 min. The cells were then cultured with Endothelial Cell Basal Medium-2 supplemented with growth factors (LONZA, MD, USA). Animals were maintained according to the institutional guidelines at the animal center of the Academy of Military Medical Sciences, and the experiment complied with the animal welfare laws. Experiment protocols were approved by the Committee for Animal Care and Use of the Academy of Military Medical Sciences.

### Transient siRNA transfection

Cells were seeded in 24-well plate at a density of 1 × 10^5^ per well in complete medium. Twenty-four hours later, cells were transfected according to the standard siRNA transfection protocol of adherent cells suggested by the manufacture (Polyplus, NY, USA). The amount of 3 μl interferin^TM^ transfection reagent was used, with the final siRNA concentration of 50 nM.

### The sequences of siRNAs and construction of stable cell line

The sequence of human siRNA against Naa10–395 was 5′-CGAGCCAUGAUAGAGAACU-3′, while the human siNaa10–464 was 5′-CCCUGCACCUCUAUUCCAA-3′. The target sequence of mouse siRNA against Naa10 was 5′-GAACAAAGGCAACGUGCUU-3′. The siRNA against human UNC5B were 5′-CGCUGCUCGACUCUAAGAA-3′ (1471) and 5′-CGCCAGAAGAUAUGCAACA-3′ (3036) respectively. Targeted sequences for shRNA-induced silencing of human and mouse siNaa10 were the same as human siNaa-464, using psilencer2.1-U6/neo plasmid. The negative control sequence of both shRNA and siRNA was 5′-UUCUCCGAACGUGUCACGU-3′. Transfection of shRNA plasmid was performed using jetPEI^TM^ transfection reagent (Polyplus, NY, USA) according to the manufacturer’s instructions. Cells were then subjected to G418 selection for another two weeks with the concentration of 400 μg/ml. The knock-down efficiency of shNaa10 induced gene silencing was monitored by western blot and real time-RT-PCR.

### Western blot analysis

Cells were homogenized in lysis buffer (50 mM Hepes pH7.5, 150 mM NaCl, 1 mM EDTA, 1% Triton X-100, 1 mM DTT, 10% glycerol and 1X protease inhibitors from Roche (Basle, Switzerland)), incubated on ice for 30 min, and then centrifuged at 15,000 g for 20 min at 4 °C. Afterwards, cell lysate was subjected to 12% SDS-PAGE and transferred to nitrocellulose membranes. The membranes were blocked with and then probed with either anti-Naa10 or anti-GAPDH antibody.

### Gene expression profile

Total RNA was extracted from H1299-shNaa10 and H1299-shRNA negative control respectively, using Trizol reagent (Invitrogen, CA, USA) according to the suggested protocol. Gene expression profiling analysis was performed using Agilent Human Gene Expression 4 × 44 K Microarray (G2519F-014850 for a slide). After normalization, the ratio of Cy3/Cy5 was used to calculate the fold changes of all the genes. Genes with fold change more than two were considered as Naa10 up-regulated or down-regulated transcripts. Gene ontology annotation was analyzed using DAVID (http://david.abcc.ncifcrf.gov/) online services. Gene expression data has been submitted to Gene Expression Omnibus (GEO) dataset (accession number: GSE87674).

### Real time-reverse transcription-PCR (Real time-RT-PCR)

Total RNA was extracted and then reverse transcribed into complementary DNA (cDNA) using Improm-II^TM^ reverse transcription kit according to the indicated protocol (Promega, Madison, WI). The reverse transcripts were amplified and quantified using ABI 7500 Real time PCR detection system (Applied Biosystems, CA, USA). Each sample contains 0.5 μl of cDNA, 0.5 μl primer (0.25 μM), and 5 μl 2x reaction mixture that included SYBR Green fluorescent dye (Promega). Results were normalized to GAPDH, and the amount of each transcript was calculated by the formula: 2^−(ΔCT(target gene)−ΔCT(GAPDH))^, Ct value means the time point when fluorescence rises appreciably above background fluorescence. Primers used for Real time-RT-PCR were listed in the supplementary Table 1.

### Phalloidin staining

Naa10 stably silenced H1299 cells were seeded on the cover slips at a density of 2 × 10^5^ cells per well in 6-well plate. Twenty-four hours later, cells were washed twice with PBS and fixed with 2% paraformaldehyde for 30 min at room temperature, followed by permeabilization with 0.5% Triton X-100 in PBS for 5 min. Cells were stained with FITC-conjugated phalloidin (Sigma-Aldrich) for 30 min according to the manufacturer’s instructions. Afterwards, cells were washed for another two times for 5 min each with PBS at room temperature. After washing, slides were mounted on 50% glycerol/PBS and imaged using a Olympus BX-51 microscope (400 × magnification).

### Tube formation

Matrigel (BD Biosciences, NJ, USA) was placed at 4 °C overnight to form liquid state, and then added to each well of a 48-well plate followed by 37 °C 30 min to allow formation of solid state. Afterwards, Naa10 stably silenced H1299 and mouse immortalized embryonic endothelial cells were plated (3 × 10^4^ cells/well) on the surface of the matrigel for 24 hours. The effect of Naa10 stably knock-down induced morphological changes on tube formation was observed microscopically and photographed at 200 × magnification.

### Cell growth studies

Forty-eight hours after siRNA transfection, a density of 2 × 10^4^ cells per well was seeded in 12-well plate for 3 days. Trypan blue (Sigma-Aldrich) with the concentration of 0.5% was used for determination of the numbers of viable cells. Cell number was counted everyday to detect the differences in growth rate between the experiment groups and the control groups. Three independent experiments were carried out. Cell growth data were reported as mean and SEM with *p*-values obtained via the paired two-way of student’s t-test.

## Results

### Naa10 was responsible for morphological changes in H1299 cell and mouse immortalized embryonic endothelial cell.

**Figure 1:**
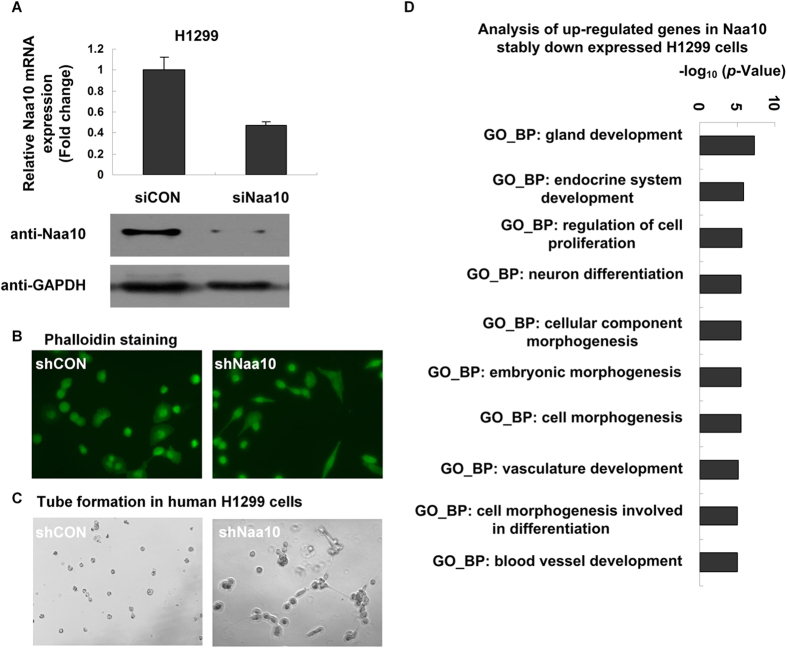
Gene expression profile exploring Naa10 gene function. (**A**) Cells were transfected with control shRNA or Naa10 shRNA for 48 hours, G418 antibiotic was then used to pool the Naa10 stably silenced cells. The knocked-down efficiency was detected by realtime-RT-PCT and western blot, and then normalized to GAPDH level. (**B**) Naa10 regulated re-organization of actin cytoskeleton was detected by FITC-conjugated phalloidin staining. Naa10 stably silenced H1299 cells were seeded on the cover slips, grown overnight, fixed, and stained with FITC-conjugated phalloidin. (**C**) Stably Naa10 down expressed H1299 cells were plated on the surface of the matrigel, *in-vitro* tube formation ability was assessed. The morphological change of tube formations was observed under a phase-contrast microscope and documented. (**D**) The up-regulated genes induced by Naa10 stably knock down in H1299 cells were analyzed with Gene ontology of biological process (GO_BP) clustering using DAVID Bioinformatics Resource 6.7. The top 10 lists were calculated by *p*-value in logarithmic, the larger the value the more meaningful prompt GO_BP.

We established Naa10 stably knocked-down H1299 cell line. Silencing efficacy of Naa10 in H1299 was verified by real time-RT-PCR and Western blot analysis (Fig. 1A). To investigate the role of Naa10 on cell morphogenesis, we performed FITC-conjugated phalloidin staining to image the actin cytoskeleton. Stable transfection of Naa10 shRNA led to significant morphological changes compared to the negative control group: Shuttle-shaped membrane protrusion was observed in shNaa10 cells, compared to the rounded-cell phenotype in the control cells (Fig. 1B). *In-vitro* tube formation assay also gave evidences to the Naa10 induced morphological change: Robust tubular-like structures were observed in shNaa10 cells in both H1299 cells and mouse immortalized embryonic endothelial cells, with no obvious tubes observed in their corresponding control group (Fig. 1C and supplementary Figure 1A). The silencing efficiency of mouse immortalized embryonic endothelial cell was shown in supplementary Figure 1B. Gene ontology analysis of Naa10 stably knocked down H1299 cell line further indicated that Naa10 is involved in negative regulation of cell morphogenesis (Fig. 1D).

### NTN1 and its receptor UNC5B were early events induced by Naa10 siRNA transiently transfection.

**Figure 2:**
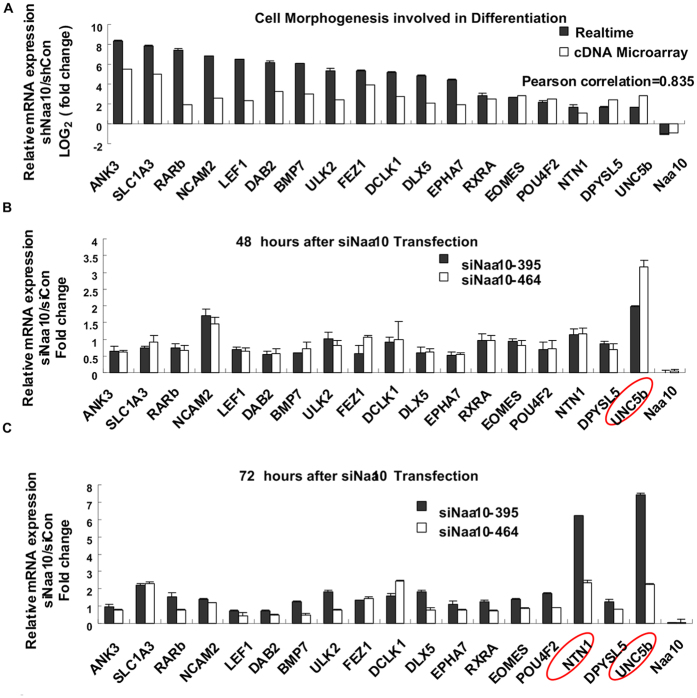
UNC5B was the early events of Naa10 stably knocked-down induced gene expression changes. (**A**) Comparison of the relative Logarithmic fold change of mRNA expressions between RNA-seq and Realtime-RT-PCR results clustered in one of the top 10 GO_BPs (cell morphogenesis involved in differentiation). Cells were stably transfected with Naa10 shRNA and its corresponding control. (**B**,**C**) Transient transfection of Naa10 siRNA and its corresponding negative control into H1299 cells to explore the driving forces of Naa10 stably knocked-down induced gene expression changes. The cells were collected at different time points (48 hours (**B**), and 72 hours (**C**) respectively), realtime-RT-PCR was then performed to detect the mRNA expression of various genes. Genes were normalized to GAPDH mRNA expression, formula of 2^−ΔΔCT^ was used to calculate the relative mRNA expression. Data was mean ± SEM, ΔCTSE < 0.2. Abbreviations are: ANK3, ankyrin 3, node of Ranvier (ankyrin G); SLC1A3, solute carrier family 1, member 3; RARb, retinoic acid receptor, beta; NCAM2, neural cell adhesion molecule 2; LEF1, lymphoid enhancer-binding factor 1; DAB2, Dab, mitogen-responsive phosphoprotein, homolog 2; BMP7, bone morphogenetic protein 7; ULK2, unc-51-like kinase 2; FEZ1, fasciculation and elongation protein zeta 1; DCLK1, doublecortin-like kinase 1; DLX5, distal-less homeobox 5; EPHA7, EPH receptor A7; RXRA, retinoid X receptor, alpha; EOMES, eomesodermin; POU4F2, POU class 4 homeobox 2; NTN1, netrin 1; DPYSL5, dihydropyrimidinase-like 5; UNC5b, unc-5 homolog B.

To explore the key genes that function as the downstream target of Naa10 induced morphological changes, the 18 genes involved in “cell morphogenesis” indicated by gene ontology analysis were further selected for further real time-RT-PCR validation. The tested genes by real time-RT-PCR were changed in the same direction compared with those of microarray data. Fold changes from real time-RT-PCR were highly correlated with those in microarray analysis (R = 0.835, p < 0.001), calculated by paired Pearson correlation (Fig. 2A). Melting-curve analysis indicated the specificity of all verified genes.

To discover the early events of Naa10 knock-down induced gene expression profile, we transiently transfected Naa10 siRNA and its corresponding negative control siRNA into H1299 cells. The cells were harvested at different time point post transfection, real time-RT-PCR was carried out to detect the fold changes in gene expression. Among the 18 genes, UNC5B was first up-regulated by transiently transfection of siNaa10 for 48 hours, followed by up-regulation of both NTN1 and UNC5B after siNaa10 transfection for 72 hours (Fig. 2B and C). These results suggested that NTN1 and its receptor UNC5B may be the early events or driving forces of the 18 up-regulated genes caused by Naa10 stably knocked-down.

### Validation the negative regulation of Naa10 towards NTN1 and UNC5B

**Figure 3:**
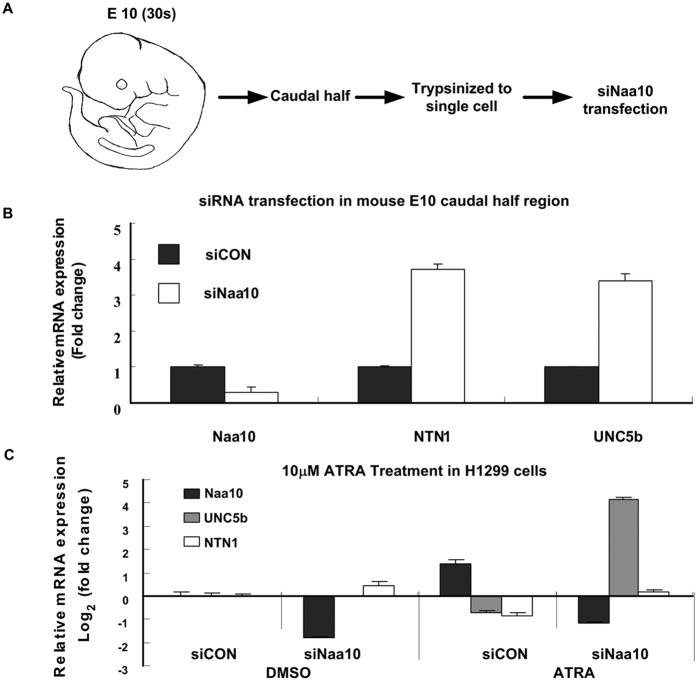
UNC5B was negatively regulated by Naa10 in E10 mouse embryos and human lung cancer cell lines. (**A**) The schematic drawing of this experiment. Obtain E10 mice embryos, separate the caudal half region and then digest into single cells, followed by transient transfection of siNaa10 to interfere its mRNA expression. (**B**) Realtime-RT-PCR was performed to detect the mRNA expression of Naa10 NTN1 and UNC5b. Data was calculated by mean ± SEM, ΔCTSE < 0.2. (**C**) Expression of Naa10 NTN1 and UNC5B upon the 10 μM ATRA and/or Naa10 siRNA treatment in non-small cell lung carcinoma cell line H1299 cells.

To investigate whether regulation pattern of Naa10 negatively regulated UNC5B mRNA expression ubiquitously existed, we separated the caudal half region of E10 mice embryos and trypsinized into single cells. Afterwards, Naa10 siRNA transfection was performed, elevated expression of NTN1 and UNC5B were detected upon siNaa10 transfection using cells from E10 mice embryos, when exuberant vasculature developed at that stage (Fig. 3A and B).

Negative regulation of Naa10 towards NTN1 and its receptor UNC5b was also discovered upon treatment of 10 uM *all-trans* retinoid acid (ATRA). As we know, retinoid acid was a known inducer of morphological differentiation^24^. Our results indicated that cells were resistant to ATRA treatment under the ATRA concentration of 1 μM and 10 μM, while ATRA concentration of 100 uM gave rise to cell growth inhibition in H1299 cells (Supplementary Figure 2A and B). We then treated the cells with 10 μM ATRA and/or Naa10 siRNA in H1299 cells, Cells were collected after 24 hours (Fig. 3C). The results showed the increased expression of Naa10 and decreased expression of both NTN1 and UNC5b upon treatment of ATRA alone, while the group treated with both siNaa10 and 10 μM ATRA reverse ATRA induced expression changes, which further solid our presumption that NTN1 and its receptor UNC5b functioned as the downstream target of Naa10 regulated genes.

## Discussion

The importance of Naa10 has been more and more recognized. Naa10 is first found to regulate cell cycle in yeast. In mammalian cells, most studies about Naa10’s function are mainly focused on its relationship with cancer. Recently, the dysfunction of Naa10 has been shown to cause Ogden Syndrome and Lenz microphthalmia syndrome^26^,^27^, which are both X-linked lethal disorder. The variety of clinical manifestations of Ogden Syndrome Lenz microphthalmia syndrome suggest that the role of Naa10 may has pleiotropic effects and affects different cellular functions.

Here we first unravel NTN1 and its receptor UNC5B as the key downstream targets of Naa10 in cancer cell line. The negative regulation was also verified in mouse caudal half region of E10 embryos. Silencing of Naa10 increases the ability of tube formation, a reflection of vasculogenesis, in both H1299 lung cancer cell line and immortalized mouse endothelial cell line. NTN1 and its receptor UNC5B function as vital genes in morphogenesis of vascular system or nervous system^20^, the negative regulation of Naa10 towards NTN1 and UNC5B may indicate a potential role of Naa10 in vasculogenesis and neurogenesis.

Some of our interesting findings in the mouse embryos can help us interpret the significance of Naa10 in the suppression of tube formation capacity. Mouse embryonic endothelial cells located in ventral dorsal aorta can develop into embryonic endothelial stem cells and hematopoietic stem cells^28^, which are two opposite cell fate decision^10^,^28^,^29^. However, faithful marker that distinguishes the transition is still unclear. Another study of ours sorted the mouse embryonic primitive endothelium into embryonic endothelial stem cells and hematopoietic stem cells. Decreased expression of Naa10 and increased tube formation capacity were identified in the endothelial stem cell population, indicating an inhibitory effect of Naa10 in vasgulogenesis.

The limitation of the present study is the lack of direct mechanism on how Naa10 regulates NTN1 and its receptor UNC5B. However, we previously demonstrated that Naa10 physically bond with RelA/p65, a NFκB transcription factor, in both colon cancer cell lines and lung cancer cell lines^30^. A negative transcriptional regulation of RelA/p65 towards NTN1 was discovered by Mirakaj *et al*. They demonstrated that upon treatment of LPS, a classical trigger of NFκB signaling pathway, a repressed expression of NTN1 were detected in wild-type mice and *in vitro* assays. RelA/p65 was then identified as a negative regulator of NTN1 by binding with NTN1 promoter region verified by chromosome immunoprecipitation (ChIP) assay^31^. From these data, we can speculate that Naa10 physically interact with p65 and negative regulate NTN1 and its receptor UNC5B.

It’s of note that we and others have reported an oncogenic role of Naa10 in diverse types of cancers. We have previously reported an oncogenic role of Naa10 concerning H1299 non-small cell lung carcinoma cell line^30^. UNC5B as the key downstream target of Naa10, we want to know whether UNC5B is contributed to the tumorigenic role of Naa10. Here, we demonstrated that Silencing of Naa10 sensitized H1299 cells to 10 μM ATRA induced cell differentiation with upregulation of UNC5B and in absence of NTN1, which is in accordance with Llambi *et al*. discovery that UNC5B induced apoptosis is fulfilled when its ligand NTN1 is absent^32^ (Fig. 3C and Supplementary Figure 2D). The above results suggested that Naa10 silencing caused inhibition of cell growth may be mediated by enhanced expression of UNC5B. To validate this hypothesis, we transfected mixed siNaa10 together with siUNC5B into H1299 cells, Naa10 siRNA was transfected for one time and UNC5B siRNA was transfected for twice. We found that knocking down of UNC5B partially rescue growth inhibition caused by silencing of Naa10 (Supplementary Figure 2E). Supplementary Figure 2F showed the interfering efficiency of Naa10 and UNC5B and the expression of NTN1 after siRNA transfection for 24 hours. These results indicated that the Naa10-UNC5B pathway may not only involves in vasculogenesis but also take part in the regulation of cell growth.

In conclusion, the study report here is the first to unravel Naa10 as the negative regulator of NTN1 and its receptor UNC5B in human lung carcinoma cell lines and caudal half region of E10 mouse embryos. Naa10 is responsible for inhibition of cell morphogenesis related tube formation ability and cell morphological change in H1299 and immortalized mouse endothelial cells. Furthermore, the Naa10 silencing induced elevated expression of NTN1 and its receptor UNC5B was also detected in caudal half region of mouse embryonic day 10. Negative regulation of Naa10 towards NTN1 and UNC5b was also detected when treating with 10uM *all-trans* retinoid acid in H1299 cells. Collectively, we reveal NTN1 and its re ceptor UNC5B as Naa10’s downstream targets, and their negative regulation may involves in regulation of both vasculogenesis and cell growth, the multifunction of Naa10 can help us better explain the variety of clinical manifestations and early death of Naa10 gene mutation induced Ogden Syndrome.

## Additional Information

**How to cite this article**: Xu, H. *et al*. Unc-5 homolog B (UNC5B) is one of the key downstream targets of N-α-Acetyltransferase 10 (Naa10). *Sci. Rep.* **6**, 38508; doi: 10.1038/srep38508 (2016).

**Publisher's note:** Springer Nature remains neutral with regard to jurisdictional claims in published maps and institutional affiliations.

## Supplementary Material

Supplementary Data
