# Supplementary material for: Unc-5 homolog B (UNC5B) is one of the key downstream targets of N-α-Acetyltransferase 10 (Naa10)
Source: Sci Rep. 2016 Dec 2;6:38508. doi: 10.1038/srep38508 (PMC5133585; doi:10.1038/srep38508)
Supplement: Supplementary Data [file srep38508-s1.doc]

**Supplementary Materials**

**Unc-5 homolog B (UNC5B) is one of the key downstream targets of N-α-Acetyltransferase 10 (Naa10)**

Huiyu Xu^1^**^*^**, Yong Han^2^**^*^**, Bing Liu^3^, Rong Li^1^

^1^Department of Obstetrics and Gynecology, Reproductive Medical Center, Peking University Third Hospital, Beijing, China

^2^Department of pathology, Zhejiang provincial people’s hospital, Hangzhou, Zhejiang Province, P. R. China

^3^307-Ivy Translational Medicine Center, Laboratory of Oncology, Affiliated Hospital of Academy of Military Medical Sciences, Beijing, China;

**^*^** HX and YH contributed equally to this paper

**Supplementary figure 1**

**The morphological change of Naa10 silenced immortalized mouse embryonic endothelial cell line.**

(A) The immortalized mouse embryonic endothelial cell line were plated on the surface of the matrigel, in-vitro tube formation ability was assessed. The morphological change of tube formations was observed under a phase-contrast microscope and documented. (B) The knocked-down efficiency of Naa10 in immortalized mouse embryonic endothelial cells was detected by realtime-RT-PCT and normalized to GAPDH mRNA level.

**Supplementary figure 1**


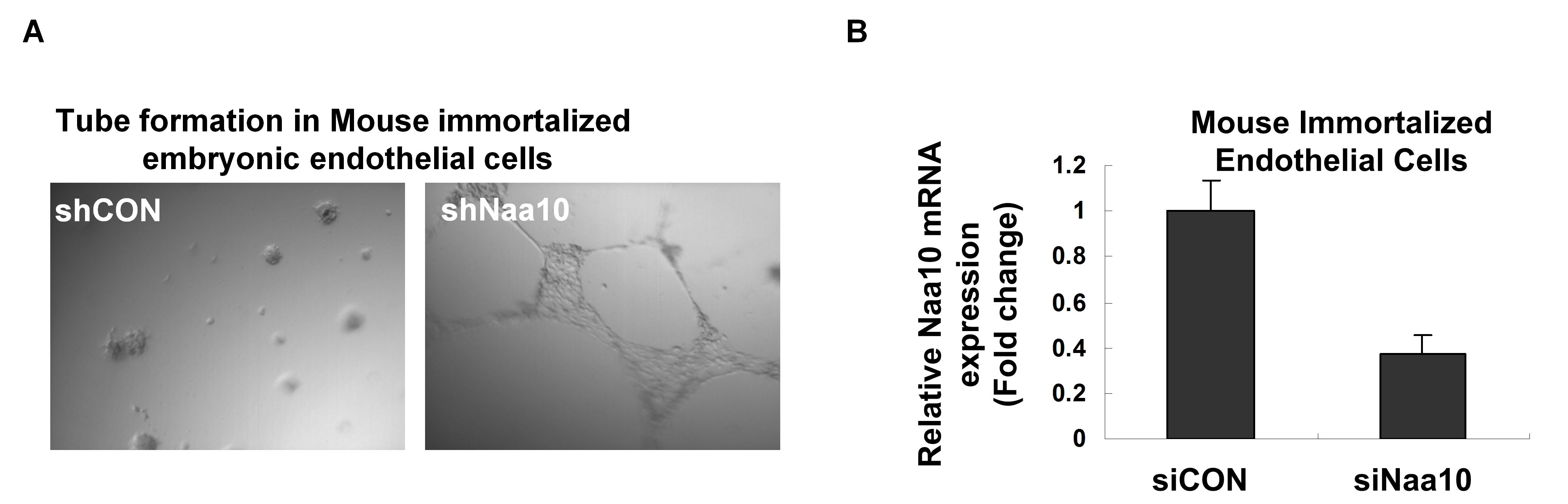


**Supplementary figure 2**

**Silencing of Naa10 sensitized H1299 cells to 10μM ATRA treatment.**

(A-C) different concentrations of ATRA treatment in H1299 cells. (D) Naa10 siRNA and its corresponding negative control siRNA were transfected into H1299 cells, 24 hours later, ATRA with the concentration of 10 uM was treated on cells for the indicated time. Live cell number was counted to examine the cell growth using trypanblue to exclude the dead. Data were mean ± SD values of triplicate experiments. (E and F) Down-expression of UNC5B partially rescue Naa10 silencing induced growth inhibition. siRNAs against both Naa10 and UNC5B with corresponding controls were transfected into H1299 cells, siNaa10 was transfected for one time, whilist the UNC5B siRNA was transfected twice. (E) Viable cells were counted to measure siRNA transfection-induced changes in cell growth. The p-Value less than 0.01 was deemed as Statistically significant (*p＜0.01) in comparison with each corresponding controls. (F) 24 hours after siRNA transfection, realtime-RT-PCR was carried out to examine the mRNA expression of Naa10, UNC5B and NTN1.

**Supplementary figure 2**

**
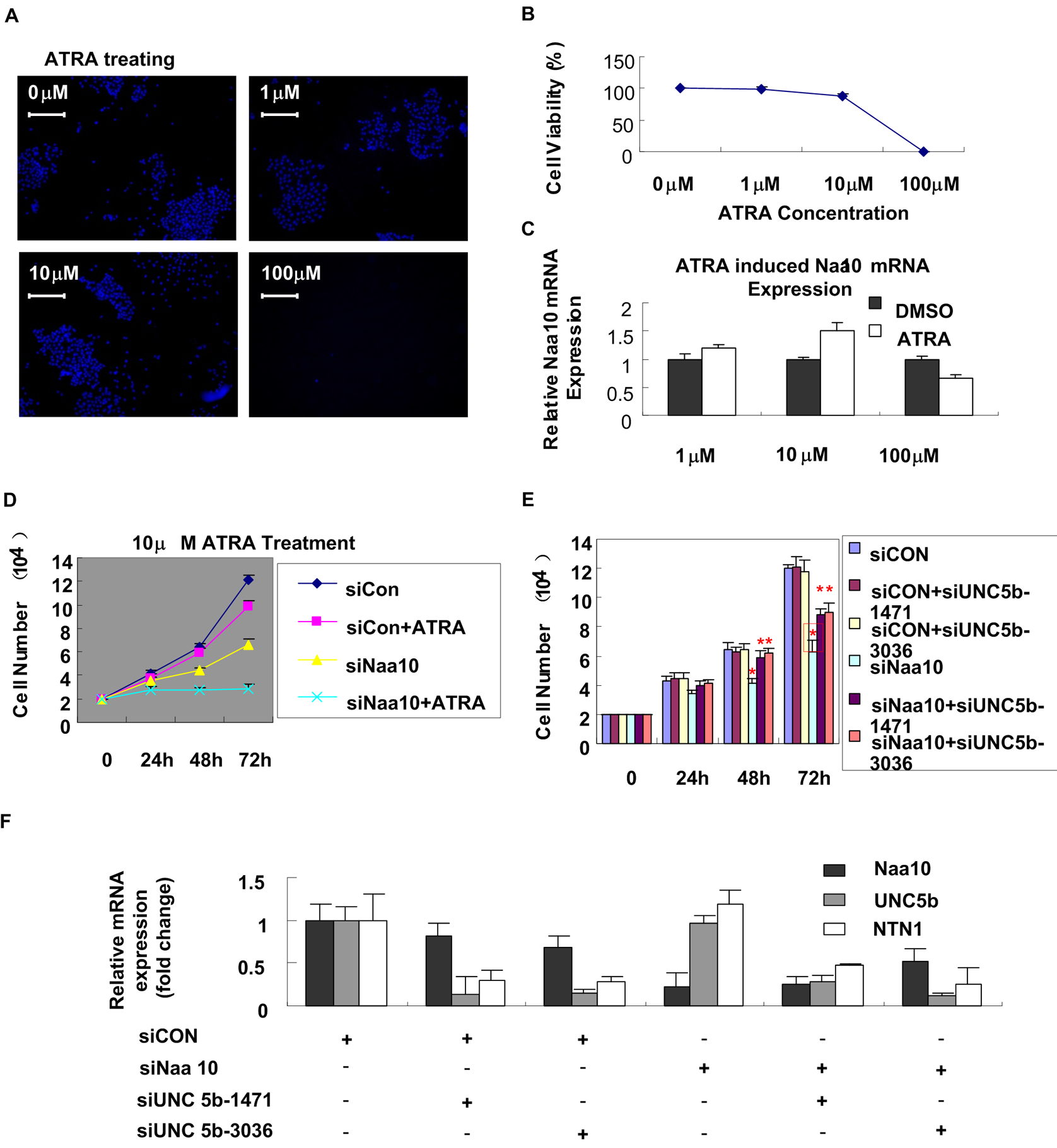
**

**Supplementary Table 1. The sequences of primers used in this article**

| h/m Naa10 | sense | Atgaacatccgcaatg |
| --- | --- | --- |
|  | anti-sense | Acaatcttcccattctc |
| hGAPDH | sense | Aaggt cggag tcaacg |
|  | anti-sense | Atggt ggtga agacgc |
| mGAPDH | sense | Catcaagaaggtggtgaagc |
|  | anti-sense | Catcgaaggtggaagagtgg |
| hRXRA | sense | Aaggttcgctaagctcttgct |
|  | anti-sense | Agcatctccataaggaaggtg |
| hDPYSL5 | sense | Aaggttagaggagtggacc |
|  | anti-sense | Agctcgctttggaacatgg |
| h SLC1A3 | sense | Agcaatggagaagagccca |
|  | anti-sense | Gtgactgtgagcagcacaa |
| hLEF1 | sense | Agcgaatgtcgttgctgagt |
|  | anti-sense | Agaccagcctggataaagct |
| hANK3 | sense | Aaccctatgtgcctgtcagt |
|  | anti-sense | Ttctccctgcttttgctcag |
| hNTN1 | sense | Accagagcctgtggatcc |
|  | anti-sense | Acgatgccgctctggtcc |
| hDAB2 | sense | Aggagaatgcagaccatga |
|  | anti-sense | Agaggcttgtgatgaacca |
| hEOMES | sense | Actaccatggacctccagaa |
|  | anti-sense | Acaagcactggtgtatactc |
| hULK2 | sense | Agccctggatgagatgtttca |
|  | anti-sense | Tgcagggtcctgtagaatcct |
| hBMP7 | sense | Atcgtgcagacgctggtc |
|  | anti-sense | Aggatgacgttggagctg |
| hFEZ1 | sense | Atcagcttcaagtccatgg |
|  | anti-sense | Tgtaactggttcttcacgg |
| hUNC5B | sense | Actttgccaccaaagcgag |
|  | anti-sense | Accagcatctcactcttgc |
| hDLX5 | sense | Aactcgccgcagtctcca |
|  | anti-sense | Aggatgcagagttctccag |
| hPOU4F2 | sense | Aacctcaagatccccggcg |
|  | anti-sense | Acttctcggcctcctcgag |
| hDCLK1 | sense | Accatgatgctgttggtcgat |
|  | anti-sense | Agctactgacagctgatgttc |
| hEPHA7 | sense | agataatttcacggcagctg |
|  | anti-sense | Agtctgaatgctgctcatga |
| hRARb | sense | Atgaacccttgaccccaagt |
|  | anti-sense | Acgagtggtgactgactgac |
| hNCAM2 | sense | Gtaacagacgtcagctgctt |
|  | anti-sense | Tacttttgccactggagcca |
| mRARb | sense | Aggtgccgaacgtgtaatta |
|  | anti-sense | Actgtgctctgctatattcc |
| mNTN1 | sense | Agagtggcatcgtggcaga |
|  | anti-sense | Tgcccttcttctcccgttg |
| mSLC1A3 | sense | Attgtcgagcacttgtccc |
|  | anti-sense | Aatc agctgatacg gcttc |
| mDCLK1 | sense | Agacgcaaccaggatgtgag |
|  | anti-sense | Gagcgaacagtctcagagga |
| mNCAM2 | sense | Caggg aaata aagaccac |
|  | anti-sense | Cagtaggctcagaatatc |
| mBMP7 | sense | Atcgtccaga cactggttca |
|  | anti-sense | Tgaca ttagagctgt cgtcg |
| mFEZ1 | sense | Tgagaagaa atcctctcc |
|  | anti-sense | Gcaaagtag gcaccttct |
| mULK2 | sense | Aatggttcaatctgcagcc |
|  | anti-sense | Gcaggatcttacttaagcc |
